# Completeness of reporting of clinical prediction models developed using supervised machine learning: a systematic review

**DOI:** 10.1186/s12874-021-01469-6

**Published:** 2022-01-13

**Authors:** Constanza L. Andaur Navarro, Johanna A. A. Damen, Toshihiko Takada, Steven W. J. Nijman, Paula Dhiman, Jie Ma, Gary S. Collins, Ram Bajpai, Richard D. Riley, Karel G. M. Moons, Lotty Hooft

**Affiliations:** 1 Julius Center for Health Sciences and Primary Care, University Medical Center Utrecht, Utrecht University, Utrecht, The Netherlands; 2 Cochrane Netherlands, University Medical Center Utrecht, Utrecht University, Utrecht, The Netherlands; 3 Center for Statistics in Medicine, NDORMS, University of Oxford, Oxford, UK; 4 NIHR Oxford Biomedical Research Centre, Oxford University Hospitals NHS Foundation Trust, Oxford, UK; 5 Centre for Prognosis Research, School of Medicine, Keele University, Keele, UK

**Keywords:** Prediction model, Diagnosis, Prognosis, Development, Validation, Reporting adherence, Reporting guideline, TRIPOD

## Abstract

**Background:**

While many studies have consistently found incomplete reporting of regression-based prediction model studies, evidence is lacking for machine learning-based prediction model studies. We aim to systematically review the adherence of Machine Learning (ML)-based prediction model studies to the Transparent Reporting of a multivariable prediction model for Individual Prognosis Or Diagnosis (TRIPOD) Statement.

**Methods:**

We included articles reporting on development or external validation of a multivariable prediction model (either diagnostic or prognostic) developed using supervised ML for individualized predictions across all medical fields. We searched PubMed from 1 January 2018 to 31 December 2019. Data extraction was performed using the 22-item checklist for reporting of prediction model studies (www.TRIPOD-statement.org). We measured the overall adherence per article and per TRIPOD item.

**Results:**

Our search identified 24,814 articles, of which 152 articles were included: 94 (61.8%) prognostic and 58 (38.2%) diagnostic prediction model studies. Overall, articles adhered to a median of 38.7% (IQR 31.0–46.4%) of TRIPOD items. No article fully adhered to complete reporting of the abstract and very few reported the flow of participants (3.9%, 95% CI 1.8 to 8.3), appropriate title (4.6%, 95% CI 2.2 to 9.2), blinding of predictors (4.6%, 95% CI 2.2 to 9.2), model specification (5.2%, 95% CI 2.4 to 10.8), and model’s predictive performance (5.9%, 95% CI 3.1 to 10.9). There was often complete reporting of source of data (98.0%, 95% CI 94.4 to 99.3) and interpretation of the results (94.7%, 95% CI 90.0 to 97.3).

**Conclusion:**

Similar to prediction model studies developed using conventional regression-based techniques, the completeness of reporting is poor. Essential information to decide to use the model (i.e. model specification and its performance) is rarely reported. However, some items and sub-items of TRIPOD might be less suitable for ML-based prediction model studies and thus, TRIPOD requires extensions. Overall, there is an urgent need to improve the reporting quality and usability of research to avoid research waste.

**Systematic review registration:**

PROSPERO, CRD42019161764.

**Supplementary Information:**

The online version contains supplementary material available at 10.1186/s12874-021-01469-6.

## Background

Clinical prediction models are used extensively in healthcare to aid patient diagnosis and prognosis of disease and health status. A diagnostic model combines multiple predictors or test results to predict the presence or absence of a certain disorder, whereas a prognostic model estimates the probability of future occurrence of an outcome [1–3]. Studies developing, validating, and updating prediction models are abundant in most clinical fields and their number will continue to increase as prediction models developed using artificial intelligence (AI) and machine learning (ML) are receiving substantial interest in the healthcare community [4].

ML, a subset of AI, offers a class of models that can iteratively learn from data, identify complex data patterns, automate model building, and predict outcomes based on what has been learned using computer-based algorithms [5, 6]. ML is often described as more efficient and accurate than conventional regression-based techniques. ML-based prediction models, correctly developed, validated, and implemented, can improve patient benefit and reduce disease and health system burden. There is increasing concern of the methodological and reporting quality of studies developing prediction models, with research till date focusing on models developed with conventional statistical techniques such as logistic and Cox regression [7–11]. Recent studies have found limited application of ML-based prediction models because of poor study design and reporting [12, 13].

Incomplete (or unclear) reporting makes ML-based prediction models difficult to interpret and impedes validation by independent researchers, thus creating barriers to their use in daily clinical practice. Complete and accurate reporting of ML-based prediction model studies will improve its interpretability, reproducibility, risk of bias assessment, and applicability in daily medical practice and is, therefore, essential for high-quality research [14]. To improve transparency and reporting of prediction model studies, the Transparent Reporting of a multivariable prediction model for Individual Prognosis Or Diagnosis (TRIPOD) Statement, a checklist of 22 items, was designed (www.tripod-statement.org) [15, 16]. Specific guidance for ML-based prediction model studies is currently lacking and has initiated the extension of TRIPOD for prediction models developed using ML or AI (TRIPOD-AI) [17, 18].

We conducted a systematic review to assess the completeness of reporting of ML-based diagnostic and prognostic prediction model studies in recent literature using the TRIPOD Statement [15, 16]. Our results will highlight specific reporting areas that can inform reporting guidelines for ML, such as TRIPOD-AI [17, 18].

## Methods

Our systematic review protocol was registered (PROSPERO, CRD42019161764) and published [19]. We reported this systematic review following the PRISMA statement [20].

### Data source and search

We searched PubMed on 19 December 2019 to identify primary articles describing prediction models (diagnostic or prognostic) using any supervised ML technique across all clinical domains published between 1 January 2018 and 31 December 2019. The search strategy is provided in the supplemental material.

### Study selection

We included articles that described the development or validation of one or more multivariable prediction models using any supervised ML technique aiming for individualized prediction of risk or outcomes. As there is still no consensus on a definition of ML, we defined a ‘study using ML’ as a study that describes the use of a non-generalized linear models to develop or validate a prediction model (e.g. tree-based models, ensembles, deep learning). Extensions to traditional statistical techniques such as generalized additive models and multivariable adaptive regression splines were considered as non-machine learning for this study. Hence, studies that claimed to have used ML, but they reported only regression-based statistical techniques were excluded from this systematic review (e.g. logistic regression, lasso regression, ridge regression and elastic net). Specifically, we focused on supervised ML, a subdomain of ML, that is characterized by the development of an algorithm that can predict (the risk of) outcomes for new observations (individuals) after learning from existing individuals and their labelled outcomes. For example, random forests, support vector machine, neural network, naïve bayes, and gradient boosting machines.

Articles reporting on the incremental value or model extension were also included. We included all articles regardless of study design, data source, or patient-related health outcome. Articles that investigated a single predictor, test or biomarker, or its causality with an outcome were excluded. Articles using ML to enhance reading of images or signals, or articles where ML models only used genetic traits or molecular markers as predictors, were also excluded. We also excluded systematic reviews, conference abstracts, tutorials, and articles for which full-text was unavailable via our institution. We restricted the search to human subjects and English-language articles. Further details are stated in our protocol [19].

Two researchers, from a group of seven (CLAN, TT, SWJN, PD, JM, RB, JAAD), independently screened titles and abstracts to identify potentially eligible studies. Full-text articles were then retrieved, and two independent researchers reviewed them for eligibility using Rayyan [21]. One researcher (CLAN) screened all articles and six researchers (TT, SWJN, PD, JM, RB, JAAD) collectively screened the same articles. Disagreements between reviewers were resolved by a third researcher (JAAD).

### Data extraction

The data extraction form was based on the TRIPOD adherence assessment form (www.tripod-statement.org) [22]. This form contains several adherence statements (hereafter called sub-items) per TRIPOD item. Some items and sub-items are applicable to all types of studies, while others are only applicable to model development only or external validation only (Table 1). To judge reporting of the requested information, sub-items were formulated to be answered with ‘yes’, ‘no’, ‘not applicable’. We amended the published adherence form by omitting the ‘referenced’ option because we checked the information in the references, supplemental material, or appendix. Sub-items 10b and 16 were extracted per model rather than at study-level, as they refer to model performance. We limited our extraction and assessment to the first model reported in the Methods section so we could achieve a consistent evaluation of the items related to the Result section as well (item 13–17).

**Table 1:** TRIPOD adherence reporting items

Reporting Items	Study design	If applicable to studies	Reporting items for TRIPOD adherence	
			Development only	Development and validation
**1. Title**	D, V		✓	✓
**2. Abstract**	D, V		✓	✓
**Introduction**				
**3. Background and objectives**				
**a.** Context and rationale	D, V		✓	✓
**b.** Objectives	D, V		✓	✓
**Methods**				
**4. Source of data**				
**a.** Source of data	D, V		✓	✓
**b.** Key dates	D, V		✓	✓
**5. Participants**				
**a.** Study setting	D, V		✓	✓
**b.** Eligibility criteria	D, V		✓	✓
**c.** Details of treatment	D, V	✓	✓	✓
**6. Outcome**				
**a.** Outcome definition	D, V		✓	✓
**b.** Blinding of outcome assessment	D,V		✓	✓
**7. Predictors**				
**a.** Predictors definition	D, V		✓	✓
**b.** Blinding of predictor assessment	D, V		✓	✓
**8. Sample size**				
Arrival at study size	D,V		✓	✓
**9. Missing Data**				
Handling of missing data	D, V		✓	✓
**10. Statistical analysis**				
**a.** Handling of predictors in the analysis	D		✓	✓
**b.** Specification of the model, all model building procedures, and internal validation methods	D		✓	✓
**c.** For validation, description of how predictions were calculated	V		✓	n.a.
**d.** Specification of all measures used to assess model performance	D, V		✓	✓
**e.** Description of model updating	V	✓	✓	n.a.
**11. Risk groups**				
Details of how risk groups were created	D, V	✓	✓	✓
**12. Development vs. validation**				
For validation, description of differences between development and validation data	V		✓	✓
**Results**				
**13. Participants**				
**a.** Flow of participants through the study	D, V		✓	✓
**b.** Description of characteristics of participants	D, V		✓	✓
**c.** For validation, comparison with development data	V		✓	✓
**14. Model development**				
**a.** Number of participants and outcome in each analysis	D		✓	✓
**b.** Unadjusted association between each candidate predictor and outcome	D	✓	✓	✓
**15. Model specification**				
**a.** Presentation of full prediction model	D	✓	✓	✓
**b.** Explanation of how to use the prediction model	D		✓	✓
**16. Model performance**				
Report of model performance measures	D,V		✓	✓
**17. Model updating**				
Results from any model updating	V	✓	✓	n.a.
**Discussion**				
**18. Limitations**				
Limitations	D, V		✓	✓
**19. Interpretation**				
**a.** For validation, interpretation of performance measure results	V			✓
**b.** Overall interpretation of results	D, V		✓	✓
**20. Implications**				
Potential clinical use of the model and implications for future research	D, V		✓	✓
**Other information**			✓	✓
**21. Supplementary information**				
Availability of supplementary resources	D, V		✓	✓
**22. Funding**				
Source of funding and role of funders	D, V		✓	✓
**Total number of applicable items for TRIPOD adherence score**			**31**	**37**

(n.a) No included studies reported external validation only or model updating (Item 10c, 10e, and 17)

We performed a double data extraction for included articles. Two reviewers independently extracted data from each article using the standardized form which was available in REDCap, a data capture tool [23]. To accomplish consistent data extraction, the form was piloted by all reviewers on five articles. One researcher (CLAN) extracted data from all articles and six researchers (TT, SWJN, PD, JM, RB, JAAD) collectively extracted data from the same articles. Discrepancies in data extraction were discussed and resolved between each pair of reviewers.

### Data synthesis and analysis

We categorized prediction model studies as prognosis or diagnosis. We also classified studies by research aim: development (with or without internal validation), development with external validation (same model), development with external validation (different model), and external validation only. Detailed definition of research aims can be found in the supplemental material. When articles described the development and/or validation of more than one prediction model, we chose the first ML model reported in the methods section for analysis.

We scored each TRIPOD item as ‘reported’ and ‘not reported’ based on answers to corresponding sub-items. If the answer to all sub-items of a TRIPOD item was scored ‘yes’ or ‘not applicable’, the corresponding item was considered ‘reported’. Two analyses were conducted: adherence per item and overall adherence per article. We calculated the adherence per TRIPOD item by dividing the number of studies that adhered to a specific item by the number of studies in which the item was applicable. The total number of TRIPOD items varies by the type of prediction model study (Table 1). We calculated the overall adherence to TRIPOD per article by dividing the sum of reported TRIPOD items by the total number of applicable TRIPOD items for each study. If an item was ‘not applicable’ for a particular study, it was excluded when calculating the overall adherence, both in the numerator and denominator [22].

Analyses were performed using R version 3.6.2 (R Core Team, Vienna, Austria). Results were summarized as percentages with confidence intervals calculated using the Wilson score interval. In addition, we also used medians, IQR ranges, and visual plots.

## Results

**Fig. 1:**
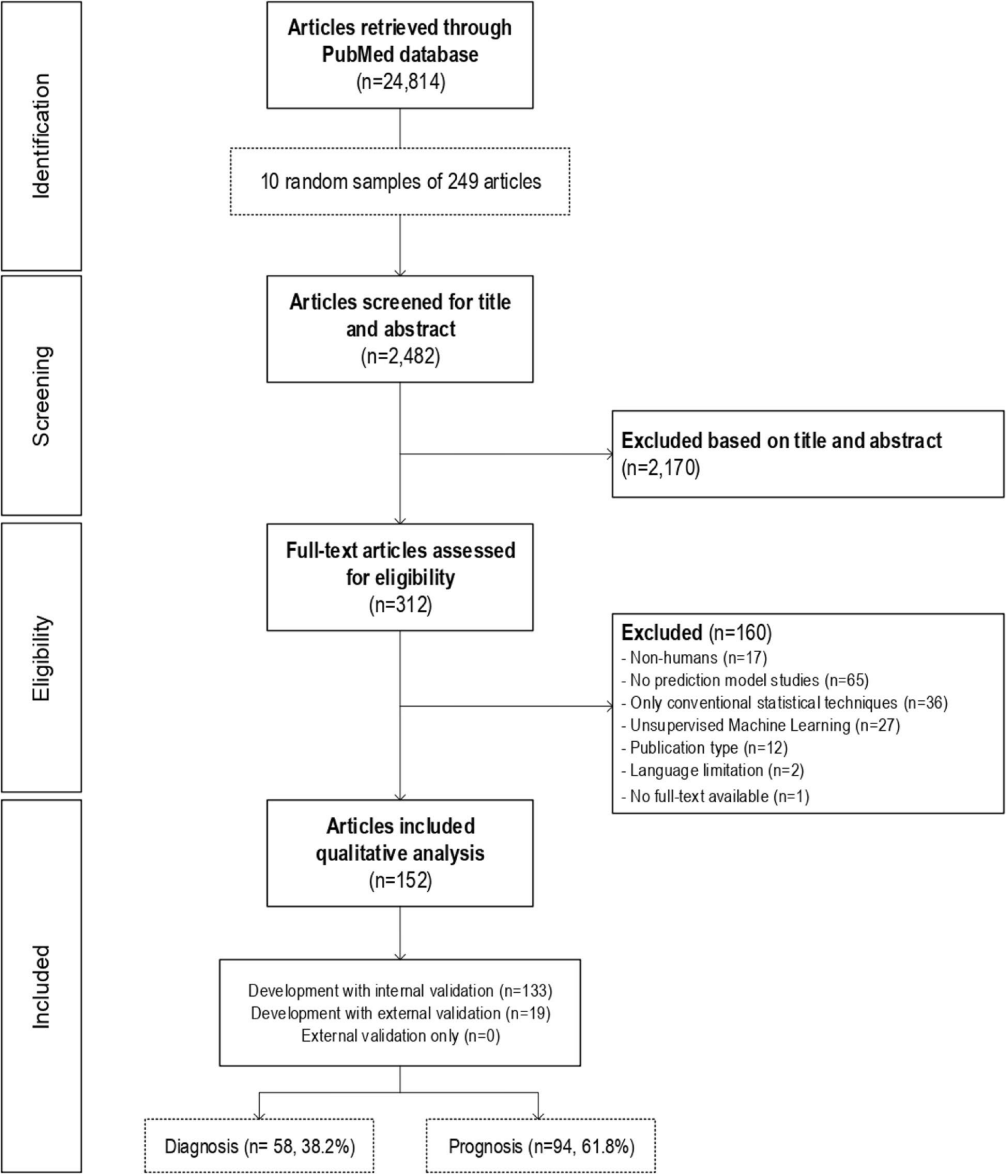
Flowchart of included studies

We identified 24,814 unique articles, of which we sampled ten random sets of 249 articles each with sampling replacement for screening. We screened title and abstracts of 2482 articles, assessed the full-text of 312 articles to finally included 152 eligible articles (Fig. 1).

We included 94 (61.8%) prognostic and 58 (38.2%) diagnostic prediction model studies. 132 (86.8%) articles described development with internal validation and 19 (12.5%) development with external validation (same model). One (0.6%) article was development with external validation (different model) and was included as a development with internal validation study in the present analysis. Prediction models were developed most often in oncology (21/152 [13.8%]). Detailed description of the included studies is provided in supplemental material.

Across the 152 studies, 1429 models were developed and 219 were validated, with a range of 1 to 156 for both types of studies. The most commonly used ML techniques for the first reported model were Classification and Regression Tree (CART [10.1%]), Support Vector Machine (SVM [9.4%]) and Random Forest (RF [9.4%]). Alongside ML techniques, 19.5% of studies reported also the development of a model using conventional statistical techniques, such as logistic regression. Five out of 152 studies (3.3, 95% CI 1.4 to 7.5) stated following the recommendations of the TRIPOD Statement.

### Overall adherence per TRIPOD item

**Fig. 2:**
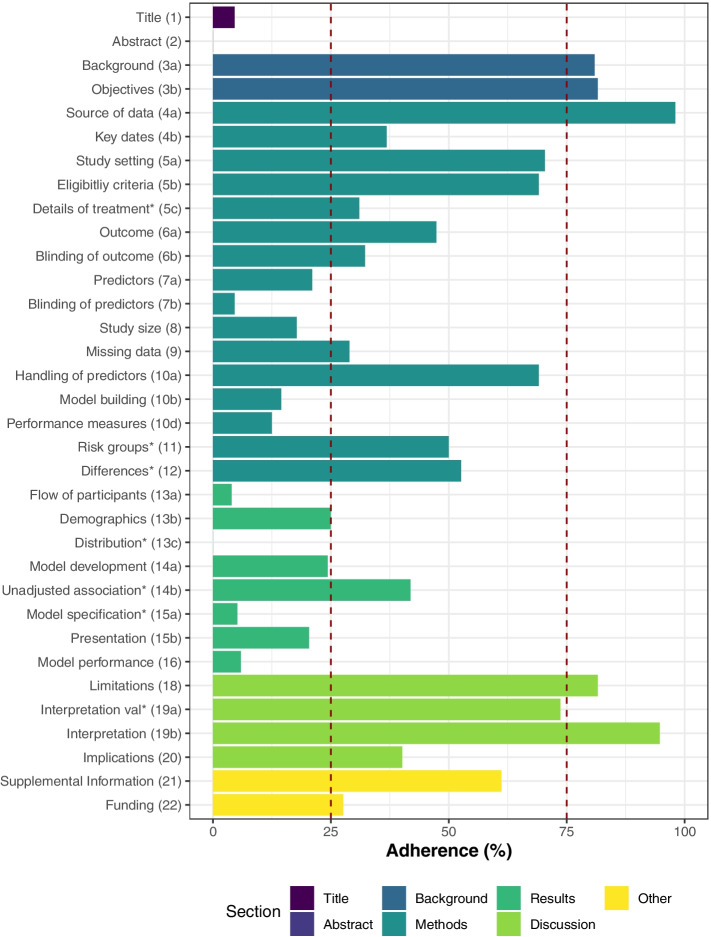
Overall adherence per TRIPOD item. Overall sample n=152

Five TRIPOD items reached at least 75% adherence (background, objectives, source of data, limitations, and interpretation), whilst 12 TRIPOD items were below 25% adherence (Fig. 2). Results for the overall adherence per TRIPOD item stratified by study type, diagnosis and prognosis, and publication year are shown in Table 2.

**Table 2:**
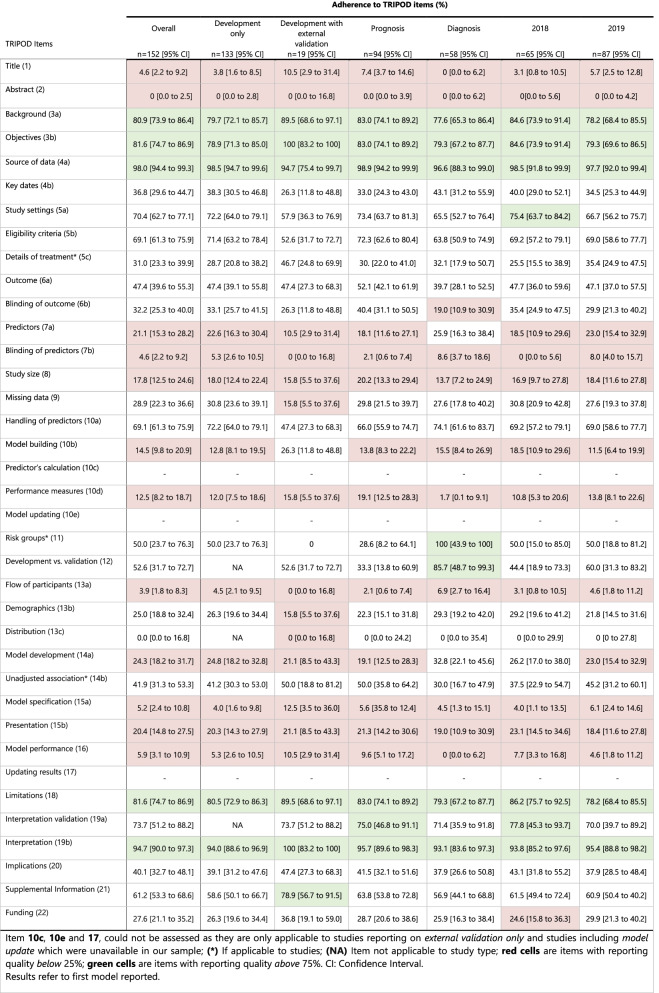
Adherence to TRIPOD items

#### Title and abstract (item 1 and 2)

Seven out of 152 studies (4.6, 95% CI 2.2 to 9.2) completely adhered to title recommendations. Description of type of prediction model study (sub-item 1.i) was poorly reported (11.2%, CI 7.0 to 17.2), but outcome to be predicted (sub-item 1.iv) was well reported (91.4%, CI 85.9 to 94.9). No study fully reported item 2, abstract (0%, CI 0 to 2.5).

#### Introduction (item 3)

Background and objectives were often reported TRIPOD items. Out of 152 studies, Background was provided in 123 studies (80.9, 95% CI 73.9 to 86.4), and the objectives were reported in 124 studies (81.6%, CI 74.6 to 86.9).

#### Methods (item 4–12)

Source of data was the most often reported item in the methods section, and across all TRIPOD items (98.0, 95% CI 94.4 to 99.3). Study setting was reported in 107/152 studies (70.4%, CI 62.7 to 77.1), eligibility criteria in 105/152 (69.1%, CI 61.3 to 75.9), and handling of predictors in 105/ 152 studies (69.1%, CI 61.3 to 75.9). Ten studies assessed risk groups and five reported complete information (50.0%, CI 23.7 to 76.3). Differences between development and validation set were reported in 10 out of 19 studies were this item was applicable (52.6%, CI 31.7 to 72.7). For 72 studies, definition of outcome was reported (47.4%, CI 39.6% to 55.3). Key study dates such as start and end date of accrual, and length of follow-up were completely reported in 56 studies (36.8%, CI 29.6 to 44.7). Details of treatment were reported in 36 out of 116 studies were this item was applicable (31.0%, CI 23.3 to 39.9). Blinding of outcome and predictors were reported in 49/152 (32.2%, CI 25.3 to 40.0) and 7/152 studies (4.6%, CI 2.2 to 9.2), respectively.

Forty-four studies reported how missing data were handled (28.9%, 95% CI 22.3 to 36.6). The missing data item consists of four sub-items of which three were rarely addressed in included studies. Within 28 studies that reported handling of missing data: three studies reported the software used (10.7%, CI 3.7 to 27.2), four studies reported the variables included in the procedure (14.3%, CI 5.7 to 31.5) and no study reported the number of imputations (0%, CI 0.0 to 39.0). Predictor definitions were given in 32/ 152 studies (21.1%, CI 15.3 to 28.2), and justification of study size was reported in 27/152 studies (17.8%, CI 12.5 to 24.6). Model building procedures, such as predictor selection and internal validation, were reported in 22/ 152 studies (14.5%, CI 9.8 to 20.9). Internal validation, a sub-item of item 10b, was one of the most reported sub-items across studies (91.4%, CI 85.9 to 94.9).

Reporting of measures used to assess and quantify the predictive performance was complete in 19 studies (12.5, 95% CI 8.2 to 18.7). Though 106/152 studies (69.7%, CI 62.0 to 76.5) reported discrimination (sub-item 10d.i), only 19/152 studies (12.5%, CI 8.2 to 18.7) reported calibration (sub-item 10d.ii). Definitions of discrimination and calibration are stated in supplemental material. Other performance measures (sub-item 10d.iii) such as sensitivity, specificity, or predictive values, were reported in 124/152 studies (81.6%, CI 74.7 to 86.9).

### Results (item 13–17)

Characteristics of study participants were reported in 38/ 152 studies (25.0, 95% CI 18.8 to 32.4). Basic demographics, at least age and gender (sub-item 13b.i), were provided in 117/152 studies (77.0%, CI 69.7 to 83.0), while summary information of the predictors (sub-item 13b.ii) was reported in 67/152 studies (44.1%, CI 36.4 to 52.0). Number of study participants with missing data for predictors (sub-item 13b.iii) was reported in 15 studies (24.2%, CI 15.2 to 36.2). Unadjusted associations were reported in 41 out of the 74 studies that reported regression-based models alongside with ML-models (41.9%, CI 31.3 to 53.3). The number of participants and events were described in 37 studies (24.3%, CI 18.2 to 31.7). In 31/ 152 studies, an explanation on how to use the developed model to make predictions for new individuals was provided, often in the form of a scoring rule or online calculator (20.4%, CI 14.8 to 27.5). Flow of participants was reported in 6/152 studies (3.9%, CI 1.8 to 8.3) and model specification was reported in 6 out of 116 studies were this item was applicable (5.2%, CI 2.4 to 10.8). Model predictive performance was completely reported in 9/ 152 studies (5.9%, CI 3.1 to 10.9).

#### Discussion (items 18–20)

Overall interpretation of results was reported in 124/152 studies (81.6, 95% CI 74.7 to 86.9). Limitations of the study were reported in 144 /152 studies (94.7, 95% CI 90.0 to 97.3). An interpretation of model performance in the validation set in comparison with the development set was given in 14/19 studies (73.7%, CI 51.2 to 88.2). Potential clinical use and implications for future research was reported in 61/152 studies (40.1%, CI 32.7 to 48.1).

#### Other information (items 21 and 22)

Availability of supplementary resources was mentioned in 93/152 studies (61.2, 95% CI 53.3 to 68.6). Funding information was reported in 42 studies (27.6%, CI 21.1 to 35.2).

### Overall adherence per article

**Fig. 3:**
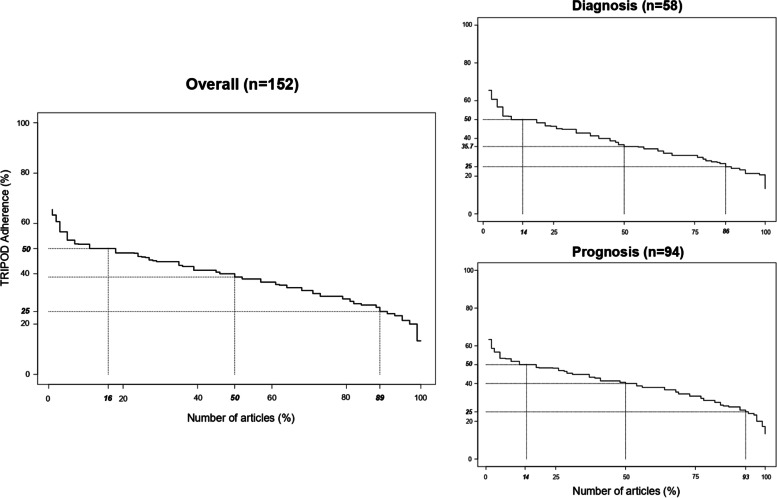
Overall adherence per article

Overall adherence of studies to items of the TRIPOD Statement ranged between 13.0 and 65.0% (median adherence =38.7% (IQR 31.0 to 46.5%)). The completeness reporting in prognostic model studies was higher (median adherence = 40.0% (IQR 33.3 to 46.8%)) than diagnostic model studies (median adherence = 35.7% (IQR 30.2 to 45.0%)) (Fig. 3). Moreover, median adherence was 40.6% (IQR 28.6 to 46.1%) in development (with internal validation) studies, compared to 37.9% (IQR 31.0 to 46.4%) in development with external validation studies.

## Discussion

We conducted a systematic review of ML-based diagnostic and prognostic prediction model studies and assessed their adherence to the TRIPOD Statement. We found that ML-based prediction model studies adhere poorly to the reporting items of the TRIPOD Statement.

Complete reporting in titles and abstracts is crucial to identify and screen articles. However, titles and abstracts were fully reported in less than 5% of articles. In addition, information about methods was infrequently reported. Complete and accurate reporting of the methods used to develop or validate a prediction model facilitates external validation, as well as replication of study results by independent researchers. For example, to enhance transparency and risk of bias assessment, it is recommended to report the number of participants with missing data and report how missing data were handled in the analysis. Handling of missing data was seldom reported, but this may be partially explained by the fact that some ML techniques can handle missing data by design (e.g. sparsity aware splitting in XGBoost and surrogate splits in decision trees) [24, 25]. Also most studies divided a single dataset into three: training, validation and test set; the last is used for internal validation. The split sample approach for internal validation was among the most reported sub-items in our sample, but several methodological studies and guidelines have long discouraged this approach [26].

Overall, most articles adhered to less than half of the applicable items considered essential for complete reporting. Authors may have avoided reporting specific details about methods and results because their objective may be to explore the data and modeling technique accuracy, rather than build models for individualized predictions in “real world” clinical settings. However, high-quality reporting is also essential for reproducibility and replication. Furthermore, most developed models were unavailable for replication, assessment, or clinical application. Only five studies referred to the TRIPOD Statement for reporting their research. Although TRIPOD was published and disseminated in 2015, it is infrequently used for reporting of ML-based prediction model studies.

We stratified studies by type (diagnosis vs prognosis), aim (development vs development with external validation), and year (2018 vs 2019). We included diagnostic model studies developed with deep learning if they used images in combination with demographic and clinical variables. Often, these studies use several numerical variables based on pixels or voxels and build prediction models based on multiple layers of statistical interaction. Both topics are challenging to report due to number of variables used and poor interpretability of interactions. This may explain why diagnostic ML-based model studies were slightly worse reported compared to prognostic studies in our sample. However, we did not observe clear differences across stratified groups as most confidence intervals overlapped.

Previous systematic reviews have shown poor reporting of regression-based prediction model studies [7, 8, 10, 11]. One study assessed the completeness of reporting in articles published in high impact journals during 2014 within 37 different clinical fields. In 146 prediction model studies, over half of TRIPOD items were not fully reported, obtaining an overall adherence of 44% (IQR 35 to 52%). Although authors excluded models using machine learning, the review found poor reporting of the title, abstract, model building, model specification and model performance, similar to our study [7]. In a sample of prediction model studies published in general medicine journals with the top 7 highest impact factor, the overall reporting adherence was 74% before, and 76% after the implementation of the TRIPOD Statement. Authors included only prediction models developed with regression techniques but also found poor reporting of model building, specification, and performance [11]. A recent study assessed the completeness of reporting of deep learning-based diagnostic model studies. Although they developed their own data extraction for reporting quality, authors found poor reporting of demographics, distribution of disease severity, patient flow, and distribution of alternative diagnosis [27]. These items were also inappropriately reported in our study with a median adherence between 0 and 47.3%. Another systematic review that assessed studies comparing the performance of diagnostic deep learning algorithms for medical imaging versus expert clinicians reported the overall adherence to TRIPOD was poor with a median of 62% (IQR 45 to 69%) [28]. In line with our results, a study about the performance of ML models showed that 68% of included articles had unclear reporting [12].

To our knowledge, this is the first systematic review evaluating the completeness of reporting of supervised ML-based prediction model studies in a broad sample of articles. We ran a validated search strategy and performed paired screening. We also used a contemporary sample of studies in our review (2018–2019). Though some eligible articles may have been missed, it is unlikely they would change the conclusions of this review. We used a systematic scoring-system enhancing the objectivity and consistency for the evaluation of adherence to a reporting guideline [22]. We used the formal TRIPOD adherence form and checklist for data extraction and assessment; however, these were developed for studies developing prediction models with regression techniques. Although we applied the option ‘not applicable’ for items that were unrelated to ML and items were excluded when calculating overall adherence, our results should be interpreted within this context.

While some items and sub-items may be less relevant for prediction models developed with ML techniques, other items are more relevant for transparent reporting in these studies. For example, source of data (4a), study size (8), missing data (9), transformation of predictors (10a.i), internal validation (10b.iv), and availability of the model (15b) acquire new relevance within the context of ML-based prediction model studies. As ML techniques are prone to overfitting, we recommend extending item 10b of the TRIPOD adherence form to include a new sub-item specifically related to penalization or shrinkage techniques. New reporting items such as the hardware (i.e. technical aspects) that was used to develop or validate an algorithm in images studies are needed, as well as data clustering. New practices such as explaining models through feature importance plot or tuning of hyper-parameters could be also added to the extension of TRIPOD for ML-based prediction models. Items such as testing of interaction terms (Item 10b-iv), unadjusted associations (14b), and regression coefficients (15a) require updating. Despite these recommendations, most TRIPOD items and sub-items are still applicable for both, regression and ML techniques and should be used to improve reporting quality.

We identified nearly 25,000 articles with prediction and ML-related terms within 2 years, similar to previous systematic reviews about deep learning models [29, 30]. The literature has become saturated with ML-based studies; thus, their identification, reporting and assessment becomes even more relevant. If studies are presented without essential details to make predictions in new patients, subsequent researchers will develop a new model, rather than validating or updating an existing model. Reporting guidelines aim to increase the transparent evaluation, replication, and translation of research into clinical practice [31]. Some reporting guidelines for ML clinical prediction models have already been developed [32, 33]. However, these guidelines are limited and do not follow the EQUATOR recommendations for developing consensus-based reporting guidelines [34]. The improvement in reporting after the introduction of a guideline has shown to be slow [31]. We acknowledge that the machine learning community developing predictive algorithm for healthcare might be unaware of the TRIPOD Statement. Improving the completeness of reporting of ML-based studies might be even more challenging given the number of techniques and associated details that need to be reported. There are also practical issues, like terminology used, word limits, or journal requirements, that are acting as barriers to complete reporting. To overcome these barriers, the use of online repositories for data, script, and complete pipeline could help researchers share their models with enough details to make predictions in new patients and to allow external validation of the model. Further journal endorsement, training, and tailored guidelines might be required to improve the completeness of reporting. Our results will provide input and support for the development of TRIPOD-AI, an initiative launched in 2019 [17, 18]. We call for a collaborative effort between algorithm developers, researchers, and journal editors to improve the adoption of good scientific practices related to reporting quality.

## Conclusion

ML-based prediction model studies currently do not adhere well to the TRIPOD reporting guideline. More than half of the TRIPOD items considered essential for transparent reporting were inadequately reported, especially regarding details of title, abstract, blinding, model building procedures, model specifications and model performance. Whilst ML brings new challenges to the development of tailored reporting guidelines, our study serves as a baseline measure to define future updates or extensions of TRIPOD tailored to ML modelling strategies.

## Supplementary Information

**Additional file 1.**

## Data Availability

The study protocol is available at doi: 10.1136/bmjopen-2020-038832 . The search strategy is available in supplemental material; detailed extracted data are available upon reasonable request to the corresponding author.
